# The Role of Dectin-2 for Host Defense Against Disseminated Candidiasis

**DOI:** 10.1089/jir.2015.0040

**Published:** 2016-04-01

**Authors:** Daniela C. Ifrim, Jessica Quintin, Flavie Courjol, Ineke Verschueren, J. Han van Krieken, Frank Koentgen, Chantal Fradin, Neil A.R. Gow, Leo A.B. Joosten, Jos W.M. van der Meer, Frank van de Veerdonk, Mihai G. Netea

**Affiliations:** ^1^ Department of Internal Medicine, Radboud Center for Infectious Diseases (RCI), Radboud University Nijmegen Medical Centre, Nijmegen, The Netherlands.; ^2^ Inserm U995, Lille, France.; ^3^ Université de Lille, Faculté de Médecine, Lille, France.; ^4^ Department of Pathology, Radboud University Nijmegen Medical Centre, Nijmegen, The Netherlands.; ^5^ Ozgene Pty Ltd, Bentley DC, Australia.; ^6^ Aberdeen Fungal Group, School of Medical Sciences, Institute of Medical Sciences, University of Aberdeen, Aberdeen, United Kingdom.

## Abstract

Despite the fact that *Candida albicans* is an important human fungal pathogen and Dectin-2 is a major pattern recognition receptor for fungi, our knowledge regarding the role of Dectin-2 for the host defense against disseminated candidiasis is limited. Dectin-2 deficient (Dectin-2^−/−^) mice were more susceptible to systemic candidiasis, and the susceptibility was mirrored by an elevated fungal load in the kidneys that correlated with the presence of large inflammatory foci. Phagocytosis of *Candida* by the macrophages lacking the Dectin-2 receptor was moderately decreased, while production of most of the macrophage-derived cytokines from Dectin-2^−/−^ mice with systemic candidiasis was decreased. No striking differences among several *Candida* mutants defective in mannans could be detected between naïve wild-type and Dectin-2^−/−^ mice, apart from the β-mannan-deficient *bmt1*Δ*/bmt2*Δ*/bmt5*Δ triple mutant, suggesting that β-mannan may partially mask α-mannan detection, which is the major fungal structure recognized by Dectin-2. Deciphering the mechanisms responsible for host defense against the majority of *C. albicans* strains represents an important step in understanding the pathophysiology of systemic candidiasis, which might lead to the development of novel immunotherapeutic strategies.

## Introduction

Although *Candida albicans* is a regular commensal of the skin, mucosal, and gut flora in susceptible individuals (Iliev and others 2012), it also represents the main cause of vaginal, mucocutaneous, and systemic candidiasis. Moreover, disseminated candidiasis has a very high mortality rate in immunocompromised patients with transplantation, chemotherapy, or extensive ICU stay (Yapar 2014). To maintain *C. albicans* in its commensal state, the human host defense system should be able to recognize the yeast and activate the immune responses to suppress the uncontrolled growth of the microorganism. This recognition is mediated by pattern recognition receptors (PRRs), which recognize pathogen-associated molecular patterns (PAMPs) of the pathogens with subsequent induction of the activation of host defense. Among the 4 PRR classes, C-type lectin receptors (CLRs) are the most important family of receptors for the recognition of *Candida spp*, mainly because the fungal cell wall is composed predominantly of polysaccharides such as α- and β-mannans (components of mannoproteins), β-glucans, and chitin (Netea and others 2008).

Dectin-2 is a CLR mostly present on dendritic cells, macrophages, and neutrophils that has been reported to recognize α-mannan (McGreal and others 2006; Saijo and others 2010), affording protection against systemic infection with *C. albicans* by inducing Th-17 immune responses (Saijo and others 2010). It signals through CARD9, resulting in subsequent NF-κB activation (Bi and others 2010). Dectin-2 forms heterodimers with Dectin-3, leading to proinflammatory responses during *C. albicans* infection (Zhu and others 2013). In addition to *C. albicans*, Dectin-2 also recognizes *Candida glabrata,* and mice lacking Dectin-2 were less able to secrete Th1 and Th17 cytokines, to produce reactive oxygen species (ROS) and to activate pathways involved in phagocytosis during activation with this pathogen (Ifrim and others 2014). Furthermore, Dectin-2 present on alveolar macrophages is involved in triggering airway inflammation mediated by the house dust mite (Clarke and others 2014), while on dendritic cells it primes the Th2 responses to this microorganism (Barrett and others 2011).

Despite these reports on the function of Dectin-2 for the recognition of fungal pathogens, only limited information is available concerning the role of Dectin-2 for triggering the innate immune responses (such as inflammation, neutrophil/macrophages phagocytosis and killing, etc) during disseminated candidiasis. In this study, we investigate the role of Dectin-2 as a PRR and an essential trigger of host defence in a murine model of systemic candidiasis. Additionally, we assessed whether α-mannans are the main fungal PAMP recognized by Dectin-2 by testing a broad panel of *Candida* cell wall mutants.

## Materials and Methods

### Generation of Dectin-2^−/−^ mice

Dectin-2^−/−^ mice were generated by Ozgene Pty Ltd, as described previously (Zhang and Lutz 2002). Eight- to 12-week-old female Dectin-2^−/−^ (*Clec4n*^−/−^) mice on a C57BL/6J background were obtained from a breeding colony at the Central Animal Laboratory, Radboud University Medical Centre and used for the experiments when they were 20–25 g. Age-matched C57BL/6J female mice were obtained from Charles River Wiga (Sulzfeld, Germany). The animals were fed standard Laboratory Chow (Hope Farms, Woerden, The Netherlands) and water *ad libitum*. The experiments were repeated at least twice with a minimum of 4 animals per time point. All experiments were approved by the Ethics Committee on Animal Experiments of Radboud University Nijmegen.

### Candida strains, culture media, and growth conditions

*C. albicans* ATCC MYA-3573 (UC 820), a strain described in detail elsewhere (Lehrer and Cline 1969), was used in all experiments. *Candida* was grown and maintained on Sabouraud dextrose (SD) plates. For inoculum preparation, a single colony was grown in SD broth at 29°C for 24 h, with shaking. Cells were washed twice in sterile phosphate-buffered saline (PBS) and counted using a hemocytometer. Cell density was adjusted with PBS to the desired inoculum level. *Candida* yeast cells were heat-killed for 30 min at 95°C. To generate hyphae, yeast cells were inoculated and grown overnight at 37°C in culture medium adjusted to pH 6.4 with hydrochloric acid. Hyphae were killed by heating at 95°C for 30 min and resuspended in culture medium to a hyphal inoculum size that originated from 1 × 10^8^ colony-forming unit (CFU)/mL.

The *OCH1* and *PMR1* genes were disrupted to generate strains with severe truncations in N- and O-linked mannan that were compared to controls strains in *och1*Δ/*och1*Δ*/OCH1* and *pmr1*Δ/*pmr1*Δ*/PMR1* in which a wild-type copy of the disrupted gene was reintroduced as described (Fonzi and Irwin 1993; Bates and others 2005, 2006). The phosphomannan-deficient *mnn4*Δ serotype B null mutant, the parental and its revertant were constructed as described (Hobson and others 2004). The *bmt1*Δ (Fabre and others 2014), *bmt2*Δ, *bmt5*Δ (Mille and others 2012), and *bmt1*Δ*/bmt2*Δ*/bmt5*Δ (Courjol and others 2015; Courjol and others 2016; Wilson and others 2000) β-mannosyl transferase mutants were constructed as previously described.

### *C. albicans* infection model and fungal burden

Wild-type and Dectin-2^−/−^ mice were injected via the lateral tail vein with *Candida* (2.5 × 10^5^ CFU per mouse) in a 100 μL volume of sterile pyrogen-free PBS. Mice were monitored daily. For survival studies, groups of 10 mice were followed up for a period of 28 days. For immunological and histological studies, a nonlethal experimental model of disseminated candidiasis was used, in which animals were injected with 1 × 10^5^ CFU per mouse via the lateral tail vein. This lower dose of *Candida* was used to avoid a bias induced by differential mortality at various time points (Netea and others 2003). Subgroups of 4 animals were sacrificed on day 3, 7, 14, or 28 after infection. Tissues were collected and processed for fungal burden and cytokine analysis. To assess the tissue outgrowth of *Candida*, the liver and kidneys were removed aseptically, weighed, and homogenized in sterile PBS in a tissue grinder. The number of viable *Candida* cells in the tissues was determined by plating serial dilutions on SD agar plates, as described elsewhere (Kullberg and others 1990). The CFUs were counted after 24 h of incubation at 29°C and expressed as log CFUs per gram of tissue.

### Histopathology

Kidney samples from wild-type and infected mice were kept in formaldehyde until processed. Sections were dehydrated with xylene, rehydrated through a graded series of ethanol solutions, and stained with hematoxylin and eosin or periodic acid-Schiff using conventional staining methods. All individual segments were evaluated for the presence and intensity of inflammation, and for the presence of fungi. Tissue sections were analyzed with a VisionTek™ digital microscope (Sakura, Japan), using VisionTek Live software.

### In vitro cytokine production

Peritoneal macrophages were isolated from mice by injecting 5 mL of ice-cold sterile PBS (pH 7.4) into the peritoneal cavity. After centrifugation and washing, cells were resuspended in RPMI-1640 culture medium containing 1 mM pyruvate, 2 mM L-glutamine, and 50 mg/L gentamicin. Cells were counted using a Z1 Coulter Particle Counter (Beckman Coulter, Woerden, The Netherlands), adjusted to 1 × 10^6^ cells/mL and cultured in 96-well round-bottom microtiter plates (Costar, Corning, The Netherlands) at 1 × 10^5^ cells/well, in a final volume of 200 μL. After 24 h of incubation with different stimuli at 37°C and 5% CO_2_, the plates were centrifuged at 1,400 *g* for 8 min, and the supernatants were collected and stored at −80°C until cytokine assays were performed.

Spleen cells were isolated by gently passing spleens through a sterile 200 μm filter chamber. After washing with sterile PBS and centrifugation at 4°C (1,200 rpm for 5 min), cells were resuspended in 2 mL RPMI-1640 in the presence of 20% fetal calf serum (FCS), counted, and concentrations were adjusted to 1 × 10^7^ cells/mL. Cells were cultured in 24-wells plates (Greiner, Alphen a/d Rijn, The Netherlands) at 5 × 10^6^ cells/well, and different stimuli were added in a final volume of 1,000 μL. Supernatants were collected at 2 different time points (depending on the cytokine) as follows: after 48 h of incubation at 37°C and 5% CO_2_, 500 μL supernatant per well were collected and stored at −80°C until cytokine assays were performed; thereafter, the plates were further incubated at 37°C and 5% CO_2_ for 3 more days. Ultimately, the plates were centrifuged at 1,400 *g* for 8 min, and the remaining supernatants were collected and stored at −80°C until cytokine assays were performed. Concentrations of mouse TNFα, IL-1α, and IL-1β were determined by specific radioimmunoassay. Mouse IL-6, KC, IL-17, IFNγ, IL-22, and IL-10 concentrations were measured by commercial ELISA kits (BioSource, Camarillo, CA), according to the instructions of the manufacturer.

### Leukocyte recruitment

Wild-type and Dectin-2^−/−^ mice were injected with 100 μL of 1 × 10^5^ heat-killed *Candida* per mouse, intraperitoneally. After 4 h cells were recruited from the peritoneum and 50 μL of cell suspension (1 × 10^6^ cells per mL) was centrifuged in a cytospin at 500 rpm for 10 min. The cells were stained with May-Grünwald Giemsa, observed and counted under the microscope.

### Phagocytosis and killing of C. albicans

Peritoneal macrophages were recruited and phagocytosis and killing assays were performed as described elsewhere (Kullberg and others 1993; Ifrim and others 2014).

### Ethics statement

All experiments in this study were carried out in strict accordance with the recommendations in the Guide for the Care and Use of Laboratory Animals of the National Institutes of Health, the Dutch law on Animal experiments, and FELASA regulations. The protocol was approved by the Ethics Committee on Animal Experiments of the Radboud University Medical Centre. All efforts were made to minimize suffering of the animals.

### Statistical analysis

Differences in phagocytosis, intracellular killing, and cytokine production were analyzed using the Mann–Whitney *U*-test. Survival data were analyzed using the Kaplan–Meyer log rank test. Differences were considered significant at *P* < 0.05. The experiments were performed at least twice. At least 4 mice per group per time point were used for the outgrowth, phagocytosis, killing, and cytokine synthesis. Ten mice/group entered the survival experiment, which was repeated 2 more times. Data are presented as mean ± SEM.

## Results

### Dectin-2^−/−^ mice are more susceptible to systemic infection with C. albicans

**FIG. 1.**
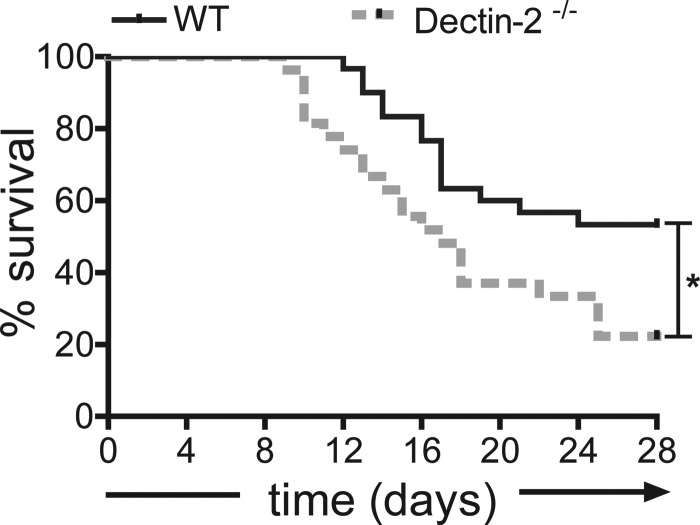
Dectin-2^−/−^ mice are more susceptible to *Candida albicans* systemic infection. Survival curves of C57BL/6J wild-type (*n* = 20, *continuous line*) and Dectin-2^−/−^ (*n* = 19, *interrupted line*) mice after intravenous injection with live *C. albicans* UC820 (2.5 × 10^5^ cells/mouse). *P = 0.012 by logrank test.

**FIG. 2.**
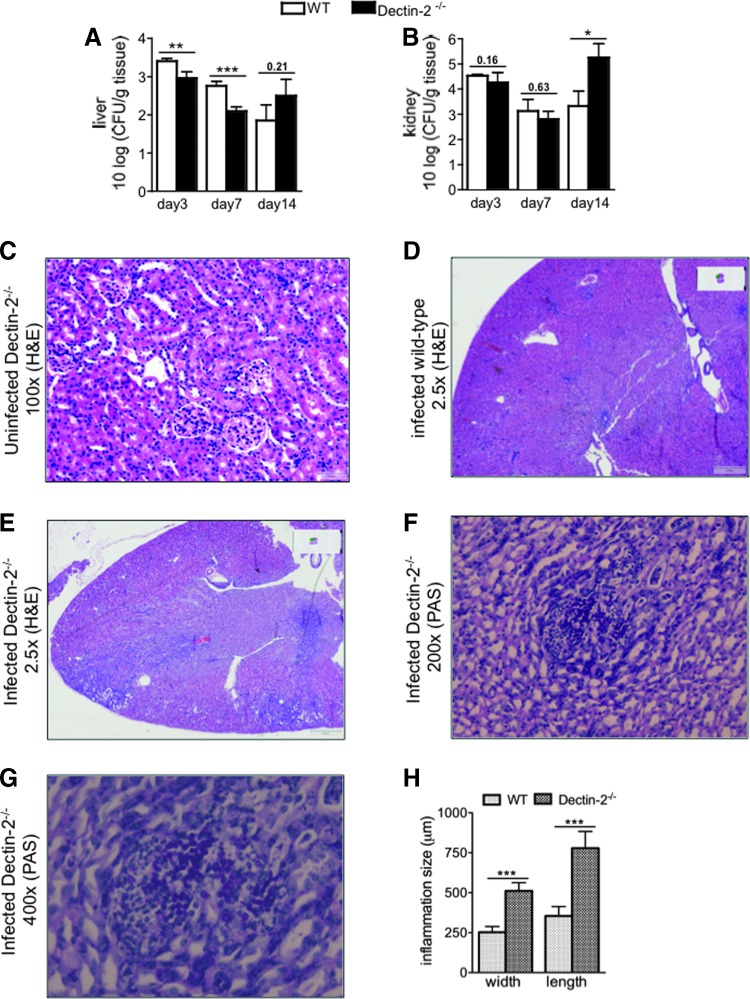
*Candida* outgrowth in the liver and kidneys of mice during systemic infection. *Candida* colony-forming units (CFUs) from liver **(A)** and kidneys **(B)** from wild-type and Dectin-2^−/−^ mice at day 3, 7, and 14 postinfection. Wild-type and Dectin-2^−/−^ mice received 1 × 10^5^ CFU live *C. albicans* (per mouse), intravenously. Results are presented as mean ± SEM (*n* = 9–12 mice per group from 3 independent experiments). Significance was determined with Mann–Whitney *U*-test. Statistically different groups are indicated as *P < 0.05; **P < 0.01; ***P < 0.001. Histopathological assessment of kidneys on day 14 in uninfected Dectin-2^−/−^ **(C,** 100×**)**, infected wild-type **(D,**2.5×**)**, infected Dectin-2^−/−^ **(E,** 2.5×**)** (hematoxylin and eosin-stained sections) and infected Dectin-2^−/−^ **(F,**200×, and **G,** 400×**)** mice (periodic acid–Schiff-stained sections). Representative localized lesions and *C. albicans* colonies are shown in the kidneys of Dectin-2^−/−^ mice **(D, E)**. The dimension of inflammatory foci is represented in **(H)**. A minimum of 20 foci were measured in at least 7 different kidneys. Results are mean ± SEM (*n* = 4 mice per group) from 2 independent experiments. Significance was determined with Mann–Whitney *U*-test. Statistically different groups are indicated as ***P < 0.001.

A lethal dose of *Candida* was intravenously injected in wild-type and Dectin-2^−/−^ mice, which were thereafter monitored for 28 days. Dectin-2^−/−^ mice had significantly lower survival than wild-type mice (Fig. 1). On day 3, 7, and 14 postinfection, organs of infected mice were isolated and CFU were counted. Interestingly, slightly fewer colonies were isolated from the livers of Dectin-2^−/−^ mice than of the wild-type mice at the early time points; however, both groups of mice were able to reduce the fungal burden in the liver (Fig. 2A). On the other hand, 100-fold more *Candida* colonies were found in the kidneys of Dectin-2^−/−^ mice than of wild-type mice at late time points postinfection (Fig. 2B), which most likely is the cause of the increased mortality in the deficient mice.

This observation was further underlined by histological examination of the kidneys of Dectin-2^−/−^ mice, which developed inflammatory foci that were significantly larger than in wild-type mice (Fig. 2C–E, H). The foci consist mainly of lymphocytes with sporadic neutrophils (Fig. 2F, G), suggesting chronic inflammation, similar to systemic infection caused by *C. glabrata* (Ifrim and others 2014). These results indicate that Dectin-2 is very important in the clearance of *Candida* predominantly from the kidneys, the target organ of disseminated candidiasis in mice.

### The impact of Dectin-2 deficiency on cytokine production capacity

**FIG. 3.**
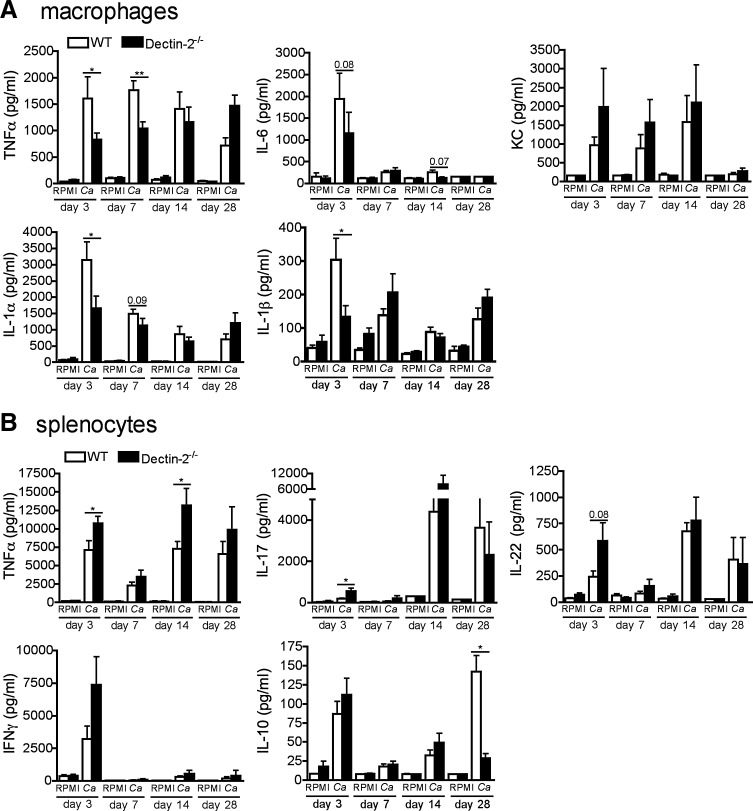
Cytokine profile during systemic infection with *C. albicans.* Wild-type or Dectin-2^−/−^ mice received 1 × 10^5^ CFU heat-killed *C. albicans* per mouse, intravenously. After 3, 7, 14, and 28 days, peritoneal macrophages **(A)** were restimulated *ex vivo* with heat-killed *C. albicans* hyphae (*Ca*) for 24 h. TNFα, IL-6, and KC accumulation in the supernatants and IL-1α and IL-1β accumulation inside the cells was measured by ELISA. Results are mean ± SEM (*n* = 8 mice per group) from 2 independent experiments. Significance was determined with Mann–Whitney *U*-test. Statistically different groups are indicated as *P < 0.001; **P < 0.01. Wild-type or Dectin-2^−/−^ mice received 1 × 10^5^ CFU live *C. albicans* per mouse, intravenously. After 3, 7, 14, and 28 days, splenocytes **(B)** were restimulated *ex vivo* with heat-killed *Ca* for 48 h and 5 days. TNFα, IL-10, IFNγ (48 h), IL-17, and IL-22 (5 days) accumulation in the supernatants was measured by ELISA. Results are mean ± SEM (*n* = 8 mice per group) from 2 independent experiments. Significance was determined with Mann–Whitney *U*-test. Statistically different groups are indicated as *P < 0.05.

For a greater understanding of immunological responses during systemic candidiasis in Dectin-2-deficient mice, we analyzed the cytokine production capacity during the course of infection with *C*. *albicans*. Interestingly, on day 3 and 7 the production of proinflammatory cytokines (TNFα, IL-6, IL-1α, and IL-1β) by macrophages of Dectin-2^−/−^ mice were significantly lower than in wild-type mice (Fig. 3A), which may represent an important cause of decreased resistance to the pathogen. On the contrary, on days 14 and 28 the production of proinflammatory cytokines in the knockout mice recovered, but this is most likely due to the increase of fungal burdens at these late time points (Fig. 3A).

We next analyzed the splenocyte-derived cytokines from mice with systemic candidiasis. Surprisingly, the production of TNFα, IL-17, and IL-22 were moderately elevated from the splenocytes of knockout mice, particularly at day 3 postinfection (Fig. 3B), while mostly leveling up during the late course of infection (Fig. 3B). Interestingly, the production of the anti-inflammatory cytokine IL-10 was significantly decreased in Dectin-2^−/−^ mice at day 28 after infection (Fig. 3B).

### The role of mannans for C. albicans recognition by Dectin-2

**FIG. 4.**
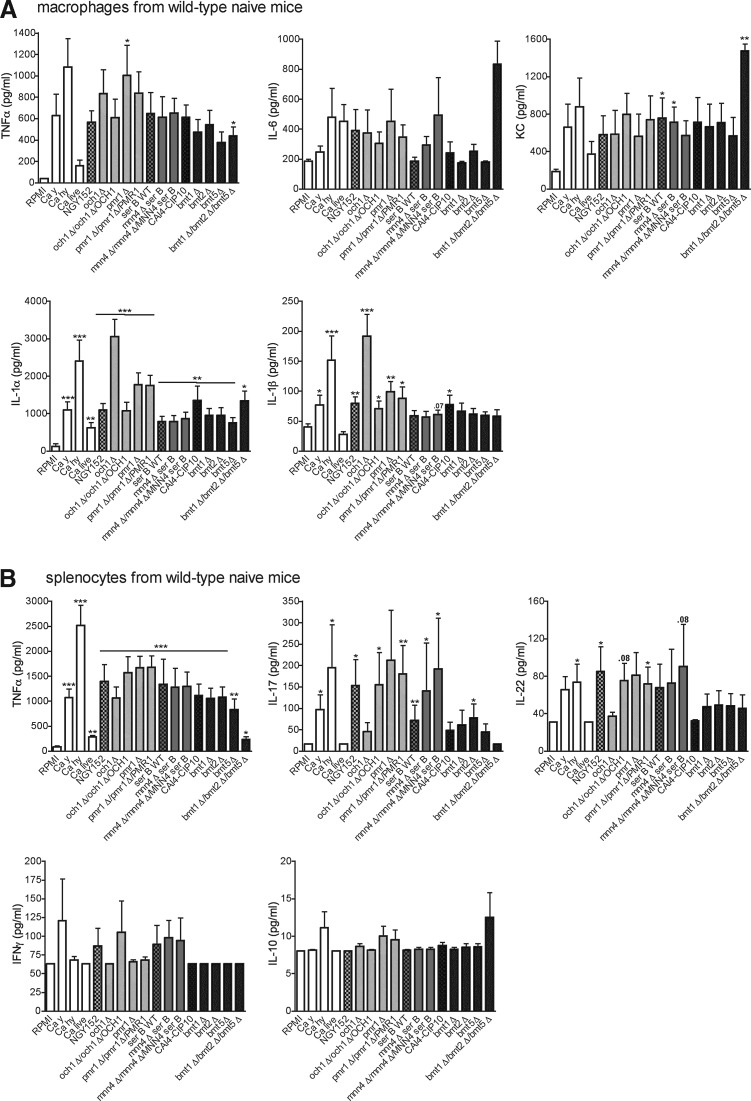
Stimulation of cells isolated from wild-type mice with *C. albicans* mutants. *C. albicans* mutants lacking *O-* and *N-*linked, α- and β-mannan were screened for their capacity to induce cytokines in wild-type peritoneal macrophages **(A)** and splenocytes **(B)** from naïve mice. Values represent mean ± SEM (*n* = 8 mice per group, 2 independent experiments). Significance was determined with Mann–Whitney *U*-test. Statistically different groups are indicated as *P < 0.05; **P < 0.01; ***P < 0.001. Ca hy, *C. albicans* hyphae heat-killed; Ca y, *C. albicans* yeast heat-killed. The control strain NGY152, was derived from CAI-4, which is the parental Ura-auxotrophic strain of all mutants analyzed, and was created by reintegration of a copy of *URA3* gene at the *RPS1* locus.

To assess whether mannans are the ligands for Dectin-2, as previously suggested in the literature (McGreal and others 2006; Saijo and others 2010), we screened several α- and β-mannan mutants for their ability to stimulate cytokine production by peritoneal macrophages and splenocytes from Dectin-2^−/−^ mice and wild-type controls. The macrophages from wild-type mice responded with normal production of TNFα, IL-6, KC, IL-1α, and IL-1β when stimulated with the majority of the *C. albicans* mutants (Fig. 4A). Interestingly, the triple mutant lacking β-mannan, *bmt1*Δ*/bmt2*Δ*/bmt5*Δ, induced significantly higher amounts of IL-6 and KC than its parental strain *C. albicans* CAI4-CIP10. A similar effect was observed for KC when peritoneal macrophages were stimulated with different concentrations of bmt1Δ/bmt2Δ/bmt5Δ *Candida* mutant (Supplementary Fig. S1; Supplementary Data are available online at www.liebertpub.com/jir). *Candida* hyphae and *och1*Δ mutant induced significant amounts of TNFα, IL-1α, and IL-1β in macrophages (Fig. 4A). Likewise, all mutants induced TNFα, IL-17 and IL-22 in splenocytes from wild-type mice, while IFNγ and IL-10 production is very low (Fig. 4B).

**FIG. 5.**
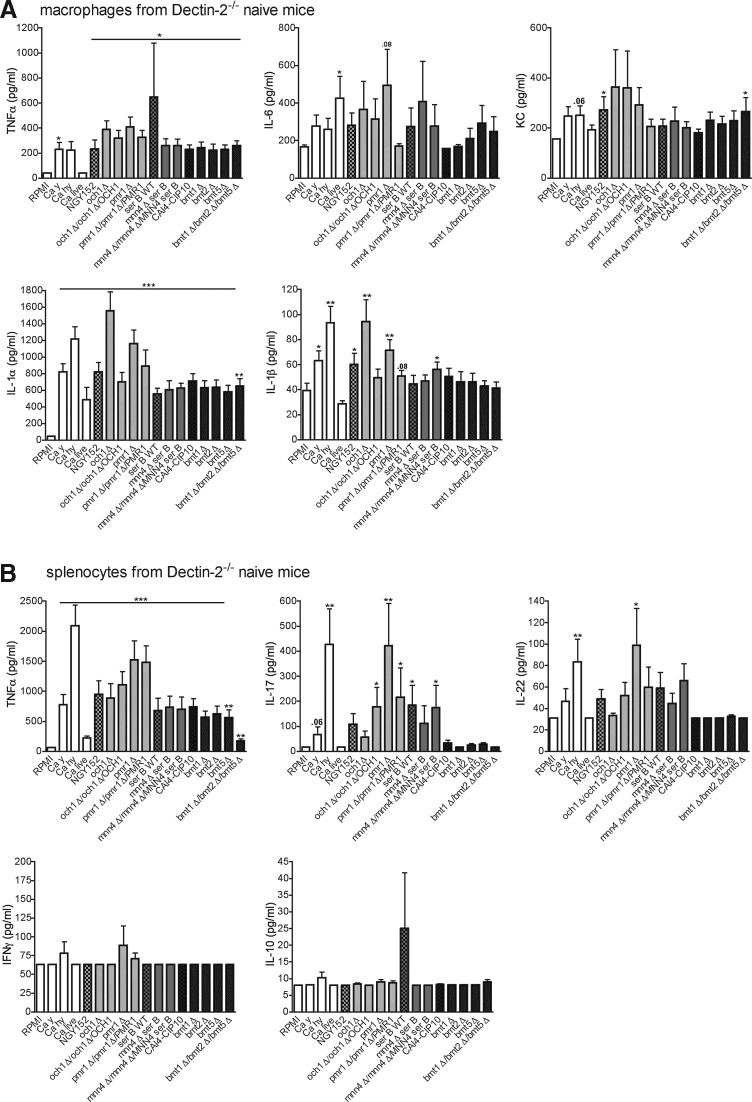
Stimulation of cells isolated from Dectin-2^−/−^ mice with *C. albicans* mutants. *C. albicans* mutants lacking *O-*, *N-*, α-, and β-mannan were screened for their capacity to induce cytokines in Dectin-2^−/−^ peritoneal macrophages **(A)** and splenocytes **(B)** from naïve mice. Values represent mean ± SEM (*n* = 8 mice per group, 2 independent experiments). Significance was determined with Mann–Whitney *U*-test. Statistically different groups are indicated as *P < 0.05; **P < 0.01; ***P < 0.001.

Peritoneal macrophages from naïve Dectin-2^−/−^ mice stimulated with *C. albicans* mutants produced, in general, lower amounts of cytokines than wild-type mice (Fig. 5A). Moreover, the triple mutant *bmt1*Δ*/bmt2*Δ*/bmt5*Δ did not induce more cytokines (in particular IL-6 and KC) than other mutants (Fig. 5A), suggesting that α-mannan ligands are better accessible for recognition by cellular receptors on macrophages, as the triple mutant only lacks β-mannosides at the terminal end of α-mannosides and phosphomannose on mannan and on phospholipomannan. Therefore, the lack of β-mannosides at the terminal end of α-mannosides suggests that α-mannans are still present on *Candida* triple mutant and, furthermore, are more exposed on *Candida* cells as they are no more hidden by β-mannosides.

### Decreased neutrophil recruitment at the site of infection in Dectin-2^−/−^ mice

**FIG. 6.**
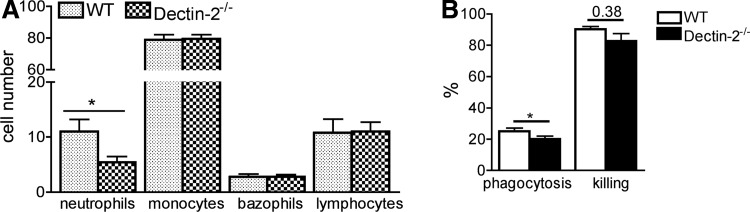
The role of Dectin-2 for leukocyte antifungal biological properties. Leukocyte recruitment in the peritoneum **(A)** wild-type and Dectin-2^−/−^ mice were injected with 100 μL of 1 × 10^5^ heat-killed *C. albicans* per mouse, intraperitoneally. After 4 h, cells were recruited, stained with May-Grünwald Giemsa, and counted under the microscope. Phagocytosis and killing of *C. albicans* cells by murine peritoneal macrophages **(B)**. The results are represented as percentage of phagocytosis (the percentage of fungal cells engulfed by macrophages present in the well) and percentage of killing (the percentage of killed fungal cells among the phagocytosed yeast). The *C. albicans*/macrophage ratio was 10:1. Values represent mean ± SEM (*n* = 5 mice per group) *P < 0.05.

Since neutrophils and monocytes are essential during fungal invasion, we investigated leukocyte recruitment after *Candida* injection into the peritoneal cavity of both Dectin-2^−/−^ and wild-type mice. The recruitment of neutrophils was decreased in knockout mice (Fig. 6A), while no differences in the recruitment of other leukocyte populations were observed. This observation, in combination with our finding of an increased level of the neutrophil chemokine KC in Dectin-2 knockout macrophages, suggests that Dectin-2 might be important for the neutrophil response to KC.

### Phagocytosis and killing of *Candida* by Dectin-2^−/−^ macrophages

Since a number of CLRs are involved in the engulfment and phagocytosis of fungi, we investigated the phagocytic capacity of peritoneal macrophages from Dectin-2 knockout mice. We consistently found a slightly reduced phagocytosis of *Candida* by the macrophages from Dectin-2^−/−^ compared to the wild-type macrophages (Fig. 6B), suggesting a role of Dectin-2 in engulfment of pathogen. The killing capacity, corrected for the decrease in phagocytosis, was not significantly decreased in macrophages of the knockout animals (Fig. 6B).

## Discussion

Our results demonstrate that mice lacking Dectin-2 are more susceptible to systemic *C. albicans* infection, and we describe the altered host defense mechanisms that are responsible for this effect. The knockout mice exhibit diminished neutrophil recruitment into the peritoneal cavity, slightly decreased phagocytosis of *Candida,* and larger inflammatory foci mostly devoid of neutrophils due to a higher fungal burden in the infected kidneys. We found that the macrophages production was lower for the majority of proinflammatory cytokines (with the exception of KC), which may represent the cause of these effects.

Our findings regarding an increased susceptibility of Dectin-2^−/−^ mice to *C. albicans* corroborate with those of Saijo and others (2010), who demonstrated similar survival curves of Dectin-2 knockout mice challenged with *C. albicans*. In addition, these findings are in line with the increased susceptibility of Dectin-2 deficient mice to *C. glabrata* (Ifrim and others 2014). Macrophage-derived proinflammatory cytokines such as TNFα, IL-1, KC, or IL-6 activate the recruitment, phagocytosis, and killing of fungi, and mice deficient in these cytokines are more susceptible to systemic candidiasis (Basu and others 2008; Gorjestani and others 2012). Interestingly, with the exception of KC, significant defects in the production of these cytokines were observed in cells isolated from *Candida*-challenged Dectin-2^−/−^ mice in our study. Saijo and others previously reported a much more dramatic decrease in cytokine production compared with the data reported here, i.e., nondetectable IL-6, TNFα, IL-1β, and IL-10 after exposure of mononuclear phagocytes to *Candida* yeasts and very low production after exposure of Dectin-2 knockout cells to hyphae. Our data show more moderate effects, which is in line with the well-known redundancy in the various PRRs responsible for the recognition of *Candida spp* (Gow and others 2012). Interestingly, the enhanced number of *Candida* colonies in the kidneys of Dectin-2^−/−^ mice correlated with enhanced proinflammatory cytokines at early time points during infection, while at late phases the synthesis of anti-inflammatory cytokines such as IL-10 became increased, most likely due to its role in the downregulation of inflammation. The fact that IL-10 production is downregulated in Dectin-2^−/−^ mice at day 28 suggests that Dectin-2 might also be important for the induction of anti-inflammatory cytokines.

Even a greater disparity with the findings of Saijo and others (2010) was found with regard to cytokine production by splenocytes. At day 14 of infection, a significant amount of IL-17 was produced by splenocytes of Dectin-2^−/−^ mice, demonstrating that the Th17 differentiation in these mice is functional. However, even though we do not exclude other (innate) cell types as source for IL-17, earlier studies of our group showed CD4^+^ is the main IL-17 producer in splenocytes (van de Veerdonk and others 2009). The increased production of cytokines by splenocytes of Dectin-2^−/−^ mice at certain time points after infection is most likely due to the higher antigen burden in the organs of the knockout mice. This is also discrepant with the findings of Saijo and others, and the cause of these differences remains to be investigated. One likely explanation may be represented by differences between various *C. albicans* strains: such differences have been earlier reported to cause differential effects of various strains for the recognition of TLR4 (Netea and others 2010; Marakalala and others 2013) and Dectin-1 (Marakalala and others 2013).

The coordinated induction of proinflammatory cytokines is important for activating multiple host defense mechanisms against fungi, including leukocyte recruitment at the site of infection, phagocytosis, and killing of the pathogen. Neutrophils are the first immune cells to migrate to the site of infection and they play a significant role in pathogen elimination through phagocytosis and killing of the microorganisms. We observed that the influx of neutrophils was decreased in the knockout mice, despite relative normal concentrations of the chemoattractant cytokine KC at early stages of infection, pointing to a role of Dectin-2 in the chemotactic response of neutrophils. However, the lack of KC production by the macrophages collected at day 28 after infection is very interesting as KC is a chemokine that induces recruitment of, mainly, neutrophils. As neutrophils characterize early infiltrates, while later on mostly monocyte-derived macrophages are attracted to the site of infection, it is tempting to speculate that this is an active regulation of different chemokine synthesis dependent on the phase of infection.

Peritoneal macrophages lacking Dectin-2 were slightly less able to ingest the pathogen, while the killing of *Candida* was not affected. Phagocytosis is the main mechanism through which immune cells engulf and clear pathogens or cell debris. The latter observation of normal *Candida* killing by Dectin-2^−/−^ cells is remarkable, since we have previously shown that Dectin-2^−/−^ neutrophils produce less ROS upon stimulation (Ifrim and others 2014), and ROS plays a significant role in *Candida* killing (Drewniak and others 2013). Drewniak and others (2013) showed that human CARD9 deficiency resulted in selective defect in the host defense against invasive fungal infection caused by an impaired phagocyte killing; this suggest that CARD9-dependent CLRs other than Dectin-2 are also important for *Candida* killing.

The increased susceptibility of Dectin-2^−/−^ mice to *C. albicans* strain UC820 is suggested to be due to their incapacity to eliminate the fungus from the kidneys, the target organ of disseminated candidiasis (Spellberg and others 2005). The fact that the animals were able to eradicate *Candida* from the liver but not kidney confirms previous data related to organ-specific *Candida* infection and immune responses mediated primarily by neutrophils and monocytes (Lionakis and others 2011; Lionakis and others 2012; Ngo and others 2014). At later time points than the 14 days assessed here it has been shown that the mice that survive completely clear the remaining *Candida*. The ultimate pathways responsible for the increased susceptibility of Dectin-2^−/−^ mice to systemic candidiasis is most likely due to the combination of defective cytokine production, reduced neutrophil recruitment, and impaired phagocytosis of the fungus.

α-mannans have been previously suggested to be the main fungal PAMP recognized by Dectin-2 (Saijo and others 2010). Furthermore, Dectin-2 binds to the terminal mannose of *N*-linked glycans (McGreal and others 2006) and recognizes mycobacterial mannose-capped lipoarabinomannan (Yonekawa and others 2014), and *O*-linked mannobiose-rich glycoproteins (α-1,2-linked mannose) from *Malassezia* (Ishikawa and others 2013). Considering these studies, we were surprised to observe that mutant *C. albicans* strains with defects in α-mannans induced largely intact cytokine amounts. However, the β-mannan-defective strains induced less cytokines in macrophages from Dectin-2^−/−^ mice compared with the wild-type animals. Interestingly, *och1*Δ and *pmr1*Δ *also* induced slightly lower Th-derived cytokines in splenocytes of wild-type and Dectin-2^−/−^ mice compared to the other strains of the fungus. Finally, the triple mutant lacking β-mannan and therefore exposing α-mannan, *bmt1*Δ*/bmt2*Δ*/bmt5*Δ, induced significantly higher amounts of IL-6 and KC than its parental strain *C. albicans* CAI4-CIP10, confirming that, indeed, *C. albicans* α-mannan ligands are better accessible for recognition by cellular receptors on macrophages. Moreover*, bmt1*Δ*/bmt2*Δ*/bmt5*Δ did not induce more cytokines than other mutants in the Dectin-2^−/−^ cells, suggesting that these exposed α-mannan ligands interact with Dectin-2.

In conclusion, we demonstrate that Dectin-2 is an important component of the anti-*Candida* host immune responses. However, the extent of immune responses triggered by Dectin-2 during systemic candidiasis may vary depending not only on *Candida* species, but also on the strain of *Candida*. Deciphering the precise mechanisms responsible for host defense against the different *C. albicans* strains represents an important step in understanding the pathophysiology of systemic candidiasis. Furthermore, revealing the exact Dectin-2 ligand(s) important for induction of antifungal host defense mechanisms could lead to the development of novel immunotherapeutic strategies and possible vaccine development.

## Supplementary Material

Supplemental data

## References

[B1] Barrett NA, Rahman OM, Fernandez JM, Parsons MW, Xing W, Austen KF, Kanaoka Y 2011 Dectin-2 mediates Th2 immunity through the generation of cysteinyl leukotrienes. J Exp Med 208(3):593–604 21357742 10.1084/jem.20100793 PMC3058587

[B2] Basu S, Quilici C, Zhang HH, Grail D, Dunn AR 2008 Mice lacking both G-CSF and IL-6 are more susceptible to *Candida albicans* infection: critical role of neutrophils in defense against *Candida albicans*. Growth Factors 26(1):23–34 18365876 10.1080/08977190801987513

[B3] Bates S, Hughes HB, Munro CA, Thomas WP, MacCallum DM, Bertram G, Atrih A, Ferguson MA, Brown AJ, Odds FC, Gow NA 2006 Outer chain N-glycans are required for cell wall integrity and virulence of *Candida albicans*. J Biol Chem 281(1):90–98 16263704 10.1074/jbc.M510360200

[B4] Bates S, MacCallum DM, Bertram G, Munro CA, Hughes HB, Buurman ET, Brown AJ, Odds FC, Gow NA 2005 *Candida albicans* Pmr1p, a secretory pathway P-type Ca2^+^/Mn2^+^-ATPase, is required for glycosylation and virulence. J Biol Chem 280(24):23408–23415 15843378 10.1074/jbc.M502162200

[B5] Bi L, Gojestani S, Wu W, Hsu YM, Zhu J, Ariizumi K, Lin X 2010 CARD9 mediates dectin-2-induced IkappaBalpha kinase ubiquitination leading to activation of NF-kappaB in response to stimulation by the hyphal form of *Candida albicans*. J Biol Chem 285(34):25969–25977 20538615 10.1074/jbc.M110.131300 PMC2923990

[B6] Clarke DL, Davis NH, Campion CL, Foster ML, Heasman SC, Lewis AR, Anderson IK, Corkill DJ, Sleeman MA, May RD, Robinson MJ 2014 Dectin-2 sensing of house dust mite is critical for the initiation of airway inflammation. Mucosal Immunol 7(3):558–567 24129160 10.1038/mi.2013.74 PMC3998635

[B7] Courjol F, Jouault T, Mille C, Hall R, Maes E, Sendid B, Mallet JM, Guerardel Y, Gow NAR, Poulain D, Fradin C 2015 β-1,2-Mannosyltransferases 1 and 3 Participate in Yeast and Hyphae *O*- and *N*-Linked Mannosylation and Alter *Candida albicans* Fitness During Infection. Open Forum Infect Dis 2(3):ofv116 26389126 10.1093/ofid/ofv116 PMC4564806

[B8] Courjol F, Mille C, Hall RA, Masset A, Aijjou R, Gow NAR, Poulain D, Jouault T, Fradin C 2016 Initiation of phospholipomannan β-1,2 mannosylation involves Bmts with redundant activity, influences its cell wall location and regulates β-glucans homeostasis but is dispensable for *Candida albicans* systemic infection. Biochimie 120:96–104 26427558 10.1016/j.biochi.2015.09.032 PMC7614791

[B9] Drewniak A, Gazendam RP, Tool AT, van Houdt M, Jansen MH, van Hamme JL, van Leeuwen EM, Roos D, Scalais E, de Beaufort C, Janssen H, van den Berg TK, Kuijpers TW 2013 Invasive fungal infection and impaired neutrophil killing in human CARD9 deficiency. Blood 121(13):2385–2392 23335372 10.1182/blood-2012-08-450551

[B10] Fabre E, Sfihi-Loualia G, Pourcelot M, Coddeville B, Krzewinski F, Bouckaert J, Maes E, Hurtaux T, Dubois R, Fradin C, Mallet JM, Poulain D, Delplace F, Guerardel Y 2014 Characterization of the recombinant *Candida albicans* beta-1,2-mannosyltransferase that initiates the beta-mannosylation of cell wall phosphopeptidomannan. Biochem J 457(2):347–360 24138199 10.1042/BJ20131012

[B11] Fonzi WA, Irwin MY 1993 Isogenic strain construction and gene mapping in *Candida albicans*. Genetics 134(3):717–728 8349105 10.1093/genetics/134.3.717 PMC1205510

[B12] Gorjestani S, Darnay BG, Lin X 2012 Tumor necrosis factor receptor-associated factor 6 (TRAF6) and TGFbeta-activated kinase 1 (TAK1) play essential roles in the C-type lectin receptor signaling in response to *Candida albicans* infection. J Biol Chem 287(53):44143–44150 23148225 10.1074/jbc.M112.414276 PMC3531730

[B13] Gow NA, van de Veerdonk FL, Brown AJ, Netea MG 2012 *Candida albicans* morphogenesis and host defence: discriminating invasion from colonization. Nat Rev Microbiol 10(2):112–122 22158429 10.1038/nrmicro2711 PMC3624162

[B14] Hobson RP, Munro CA, Bates S, MacCallum DM, Cutler JE, Heinsbroek SE, Brown GD, Odds FC, Gow NA 2004 Loss of cell wall mannosylphosphate in *Candida albicans* does not influence macrophage recognition. J Biol Chem 279(38):39628–39635 15271989 10.1074/jbc.M405003200

[B15] Ifrim DC, Bain JM, Reid DM, Oosting M, Verschueren I, Gow NA, van Krieken JH, Brown GD, Kullberg BJ, Joosten LA, van der Meer JW, Koentgen F, Erwig LP, Quintin J, Netea MG 2014 Role of Dectin-2 for host defense against systemic infection with *Candida glabrata*. Infect Immun 82(3):1064–1073 24343653 10.1128/IAI.01189-13 PMC3957982

[B16] Iliev ID, Funari VA, Taylor KD, Nguyen Q, Reyes CN, Strom SP, Brown J, Becker CA, Fleshner PR, Dubinsky M, et al. 2012 Interactions between commensal fungi and the C-type lectin receptor Dectin-1 influence colitis. Science 336(6086):1314–1317 22674328 10.1126/science.1221789 PMC3432565

[B17] Ishikawa T, Itoh F, Yoshida S, Saijo S, Matsuzawa T, Gonoi T, Saito T, Okawa Y, Shibata N, Miyamoto T, Yamasaki S 2013 Identification of distinct ligands for the C-type lectin receptors Mincle and Dectin-2 in the pathogenic fungus Malassezia. Cell Host Microbe 13(4):477–488 23601109 10.1016/j.chom.2013.03.008

[B18] Kullberg BJ, van ’t Wout JW, Hoogstraten C, van Furth R 1993 Recombinant interferon-gamma enhances resistance to acute disseminated *Candida albicans* infection in mice. J Infect Dis 168(2):436–443 8335982 10.1093/infdis/168.2.436

[B19] Kullberg BJ, van ’t Wout JW, van Furth R 1990 Role of granulocytes in increased host resistance to *Candida albicans* induced by recombinant interleukin-1. Infect Immun 58(10):3319–3324 2144844 10.1128/iai.58.10.3319-3324.1990 PMC313656

[B20] Lehrer RI, Cline MJ 1969 Interaction of *Candida albicans* with human leukocytes and serum. J Bacteriol 98(3):996–1004 4182532 10.1128/jb.98.3.996-1004.1969 PMC315286

[B21] Lionakis MS, Fischer BG, Lim JK, Swamydas M, Wan W, Richard Lee CC, Cohen JI, Scheinberg P, Gao JL, Murphy PM 2012 Chemokine receptor Ccr1 drives neutrophil-mediated kidney immunopathology and mortality in invasive candidiasis. PLoS Pathog 8(8):e1002865 22916017 10.1371/journal.ppat.1002865 PMC3420964

[B22] Lionakis MS, Lim JK, Lee CC, Murphy PM 2011 Organ-specific innate immune responses in a mouse model of invasive candidiasis. J Innate Immun 3(2):180–199 21063074 10.1159/000321157 PMC3072204

[B23] Marakalala MJ, Vautier S, Potrykus J, Walker LA, Shepardson KM, Hopke A, Mora-Montes HM, Kerrigan A, Netea MG, Murray GI, Maccallum DM, Wheeler R, Munro CA, Gow NA, Cramer RA, Brown AJ, Brown GD 2013 Differential adaptation of *Candida albicans in vivo* modulates immune recognition by dectin-1. PLoS Pathog 9(4):e1003315 23637604 10.1371/journal.ppat.1003315 PMC3630191

[B24] McGreal EP, Rosas M, Brown GD, Zamze S, Wong SY, Gordon S, Martinez-Pomares L, Taylor PR 2006 The carbohydrate-recognition domain of Dectin-2 is a C-type lectin with specificity for high mannose. Glycobiology 16(5):422–430 16423983 10.1093/glycob/cwj077

[B25] Mille C, Fradin C, Delplace F, Trinel PA, Masset A, Francois N, Coddeville B, Bobrowicz P, Jouault T, Guerardel Y, Wildt S, Janbon G, Poulain D 2012 Members 5 and 6 of the *Candida albicans* BMT family encode enzymes acting specifically on beta-mannosylation of the phospholipomannan cell-wall glycosphingolipid. Glycobiology 22(10):1332–1342 22745283 10.1093/glycob/cws097

[B26] Netea MG, Brown GD, Kullberg BJ, Gow NA 2008 An integrated model of the recognition of *Candida albicans* by the innate immune system. Nat Rev Microbiol 6(1):67–78 18079743 10.1038/nrmicro1815

[B27] Netea MG, Gow NA, Joosten LA, Verschueren I, van der Meer JW, Kullberg BJ 2010 Variable recognition of *Candida albicans* strains by TLR4 and lectin recognition receptors. Med Mycol 48(7):897–903 20166865 10.3109/13693781003621575

[B28] Netea MG, Vonk AG, van den Hoven M, Verschueren I, Joosten LA, van Krieken JH, van den Berg WB, Van der Meer JW, Kullberg BJ 2003 Differential role of IL-18 and IL-12 in the host defense against disseminated *Candida albicans* infection. Eur J Immunol 33(12):3409–3417 14635050 10.1002/eji.200323737

[B29] Ngo LY, Kasahara S, Kumasaka DK, Knoblaugh SE, Jhingran A, Hohl TM 2014 Inflammatory monocytes mediate early and organ-specific innate defense during systemic candidiasis. J Infect Dis 209(1):109–119 23922372 10.1093/infdis/jit413 PMC3864383

[B30] Saijo S, Ikeda S, Yamabe K, Kakuta S, Ishigame H, Akitsu A, Fujikado N, Kusaka T, Kubo S, Chung SH, Komatsu R, Miura N, Adachi Y, Ohno N, Shibuya K, Yamamoto N, Kawakami K, Yamasaki S, Saito T, Akira S, Iwakura Y 2010 Dectin-2 recognition of alpha-mannans and induction of Th17 cell differentiation is essential for host defense against *Candida albicans*. Immunity 32(5):681–691 20493731 10.1016/j.immuni.2010.05.001

[B31] Spellberg B, Ibrahim AS, Edwards JE Jr, Filler SG 2005 Mice with disseminated candidiasis die of progressive sepsis. J Infect Dis 192(2):336–343 15962230 10.1086/430952

[B32] van de Veerdonk FL, Marijnissen RJ, Kullberg BJ, Koenen HJ, Cheng SC, Joosten I, van den Berg WB, Williams DL, van der Meer JW, Joosten LA, Netea MG 2009 The macrophage mannose receptor induces IL-17 in response to *Candida albicans*. Cell Host Microbe 5(4):329–340 19380112 10.1016/j.chom.2009.02.006

[B33] Wilson RB, Davis D, Enloe BM, Mitchell AP 2000 A recyclable *Candida albicans* URA3 cassette for PCR product-directed gene disruptions. Yeast 16(1):65–70 10620776 10.1002/(SICI)1097-0061(20000115)16:1<65::AID-YEA508>3.0.CO;2-M

[B34] Yapar N 2014 Epidemiology and risk factors for invasive candidiasis. Ther Clin Risk Manag 10:95–105 24611015 10.2147/TCRM.S40160 PMC3928396

[B35] Yonekawa A, Saijo S, Hoshino Y, Miyake Y, Ishikawa E, Suzukawa M, Inoue H, Tanaka M, Yoneyama M, Oh-Hora M, Akashi K, Yamasaki S 2014 Dectin-2 is a direct receptor for mannose-capped lipoarabinomannan of mycobacteria. Immunity 41(3):402–413 25176311 10.1016/j.immuni.2014.08.005

[B36] Zhang Z, Lutz B 2002 Cre recombinase-mediated inversion using lox66 and lox71: method to introduce conditional point mutations into the CREB-binding protein. Nucleic Acids Res 30(17):e90 12202778 10.1093/nar/gnf089 PMC137435

[B37] Zhu LL, Zhao XQ, Jiang C, You Y, Chen XP, Jiang YY, Jia XM, Lin X 2013 C-type lectin receptors Dectin-3 and Dectin-2 form a heterodimeric pattern-recognition receptor for host defense against fungal infection. Immunity 39(2):324–334 23911656 10.1016/j.immuni.2013.05.017

