# Supplementary material for: The Role of Dectin-2 for Host Defense Against Disseminated Candidiasis
Source: J Interferon Cytokine Res. 2016 Apr 1;36(4):267–76. doi: 10.1089/jir.2015.0040 (PMC4827303; doi:10.1089/jir.2015.0040)
Supplement: Supplemental data [file Supp_Figure1.pdf]

Supplementary Data

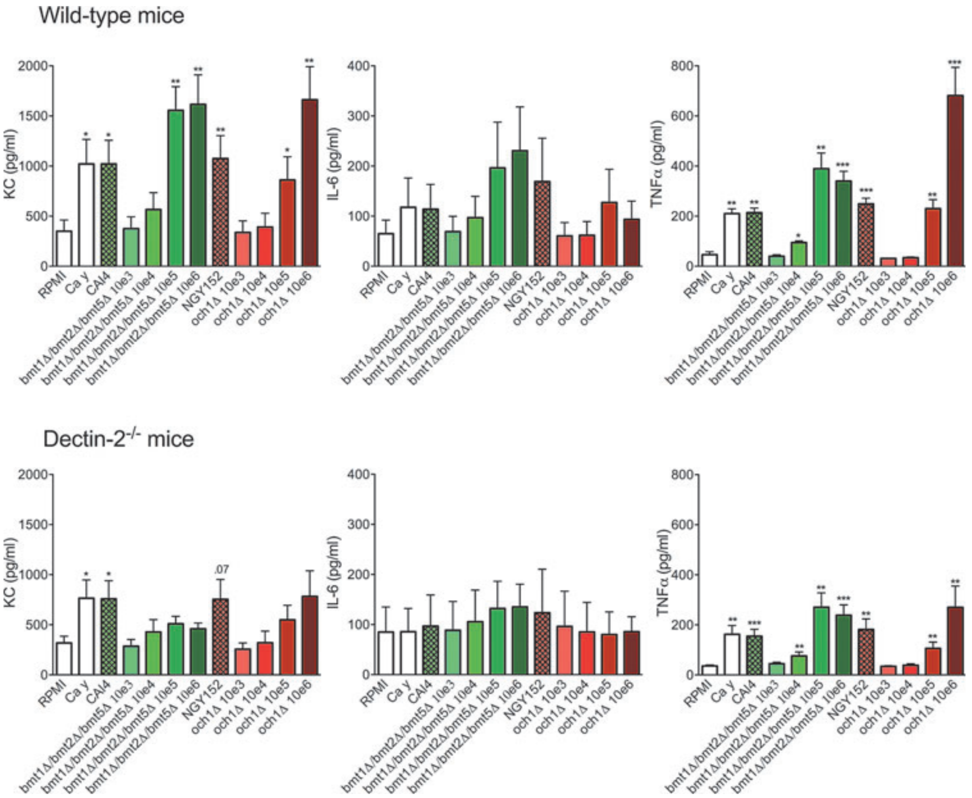

SUPPLEMENTARY FIG. S1. Stimulation of cells isolated from wild-type and Dectin-2$^{-/-}$ mice with different concentrations of *Candida albicans* mutants. Peritoneal macrophages from naïve wild-type and naïve knockout mice were stimulated with several concentrations of *C. albicans bmt1/bmt2/bmt5* and *och1* mutants and their parental strains. Values represent mean ± SEM ($n = 8$ mice per group). Significance was determined with Mann–Whitney *U*-test. Statistically different groups are indicated as *$P < 0.05$; **$P < 0.01$; ***$P < 0.001$. Ca y, *C. albicans* yeast heat-killed.
